# GRB10 and E2F3 as Diagnostic Markers of Osteoarthritis and Their Correlation with Immune Infiltration

**DOI:** 10.3390/diagnostics10030171

**Published:** 2020-03-22

**Authors:** Ya-Jun Deng, En-Hui Ren, Wen-Hua Yuan, Guang-Zhi Zhang, Zuo-Long Wu, Qi-Qi Xie

**Affiliations:** Key Laboratory of Orthopaedics Disease of Gansu Province, Lanzhou University, Lanzhou 730000, China; dengyajun205@163.com (Y.-J.D.); reneh17@lzu.edu.cn (E.-H.R.); yuanwh17@lzu.edu.cn (W.-H.Y.); zhanggzh18@lzu.edu.cn (G.-Z.Z.); wuzl18@lzu.edu.cn (Z.-L.W.)

**Keywords:** osteoarthritis, immune cells, diagnostic, Gene Expression Omnibus, CIBERSORT

## Abstract

This study aimed to find potential diagnostic markers for osteoarthritis (OA) and analyze the role of immune cells infiltration in this pathology. We used OA datasets from the Gene Expression Omnibus database. First, R software was used to identify differentially expressed genes (DEGs) and perform functional correlation analysis. Then least absolute shrinkage and selection operator (LASSO) logistic regression and support vector machine-recursive feature elimination algorithms were used to screen and verify the diagnostic markers of OA. Finally, CIBERSORT was used to evaluate the infiltration of immune cells in OA tissues, and the correlation between diagnostic markers and infiltrating immune cells was analyzed. A total of 458 DEGs were screened in this study. GRB10 and E2F3 (AUC = 0.962) were identified as diagnostic markers of OA. Immune cell infiltration analysis found that resting mast cells, T regulatory cells, CD4 memory resting T cells, activated NK cells, and eosinophils may be involved in the OA process. In addition, GRB10 was correlated with NK resting cells, naive CD4 + T cells, and M1 macrophages, while E2F3 was correlated with resting mast cells. In conclusion, GRB10 and E2F3 can be used as diagnostic markers of osteoarthritis, and immune cell infiltration plays an important role in the occurrence and progression of OA.

## 1. Introduction

Osteoarthritis (OA) is one of the most common joint diseases, especially in the elderly, with approximately 3 million newly diagnosed cases each year [1]. OA is characterized by cartilage degradation, synovial inflammation, subchondral bone remodeling, and osteophyte formation, which ultimately leads to joint function loss [2]. Therefore, OA is considered to be one of the main causes of disability worldwide [3]. Currently, the routine diagnosis of OA is usually based on clinical manifestations and joint imaging techniques; thus, a precise early diagnosis of OA is not possible [4]. Because the diagnosis is not established early, the disease progresses for most patients resulting in a poor prognosis and ineffective treatment options. Therefore, exploring biomarkers that would make an early diagnosis possible is crucial for improving the prognosis of OA patients.

In recent years, more and more studies have shown that immune cell infiltration plays an important role in the occurrence and development of OA. For example, OA joints have been shown to exhibit a distinct pattern of CD4 + T cell infiltration. CD4 + T cells promote the polarization of activated Th1 cells and increase the secretion of immunoregulatory cytokines. This local inflammation further exacerbates the OA process [5]. Moradi et al. [6] showed that OA joints present with immune cell infiltration, including CD14 + macrophages, CD4 + T cells, CD8 + T cells, and CD16 + CD56 + natural killer cells. Therefore, from the perspective of the immune system, assessing the infiltration of immune cells and determining the differences in the components of infiltrating immune cells are of great value to clarifying the molecular mechanism underlying OA and developing new immunotherapeutic targets. CIBERSORT is an analysis tool that uses RNA-seq data to evaluate the expression of immune cells and obtain various immune cell proportions from samples [7]. It has been widely used in the analysis of immune cell infiltration in a variety of diseases such as lupus nephritis [8], atopic dermatitis [9], and colorectal cancer [10]. However, so far, no studies have used CIBERSORT to analyze immune cell infiltration in OA.

In this study, we first downloaded the microarray dataset of OA from the Gene Expression Omnibus (GEO) database and performed differential expression gene analysis and then used machine learning algorithms to further screen and determine the diagnostic markers of OA. Subsequently, we used CIBERSORT for the first time to analyze the difference in immune infiltration between OA tissues and normal tissues in 22 immune cell subsets. In addition, we studied the relationship between diagnostic markers and infiltrating immune cells to better understand the molecular immune mechanism during the development of OA.

## 2. Materials and Methods 

### 2.1. Data Download

We used the “*GEOquery*” package [11] of R software (version 3.6.1, http://r-project.org/) to download the OA expression profile datasets GSE55235 [12], GSE55457 [12], and GSE51588 [13] from the GEO (https://www.ncbi.nlm.nih.gov/geo/) database [14].

### 2.2. Data Preprocessing and Differentially Expressed Genes (DEGs) Screening

Raw data of GSE55235, GSE55457, and GSE51588 datasets were read through the “*affy*” package [15], and the RMA algorithm was used for background correction and data normalization. The GSE55235 and GSE55457 gene expression matrices were then combined, and the inter-batch difference was removed using the “*sva*” package [16]. The quantile–quantile plot (Q–Q plot) was used to visualize the effect of removing inter-batch difference, and the effect of inter-sample correction was demonstrated using a two-dimensional PCA cluster plot. DEGs were screened by the “*limma*” package [17], and a volcano map of DEGs was drawn using the “*ggplot2*” package [18] to show the differential expression of DEGs. DEGs with *p* < 0.05 and |log_2_FC|>1 were considered statistically significant.

### 2.3. Functional Correlation Analysis

We used the “*clusterProfiler*” package [19] to perform Gene Ontology (GO) and Disease Ontology (DO) enrichment analyses on DEGs, respectively. Gene set enrichment analysis (GSEA) was performed on the gene expression matrix through the “*clusterProfiler*” package and “c2.cp.kegg.v7.0.symbols.gmt” was selected as the reference gene set. A false discovery rate (FDR) < 0.25 and *p* < 0.05 were considered significant enrichment.

### 2.4. Screening and Verification of Diagnostic Markers

We used least absolute shrinkage and selection operator (LASSO) logistic regression [20] and support vector machine-recursive feature elimination (SVM-RFE) [21] to perform feature selection to screen diagnostic markers for OA. The expression matrices of the GSE55235, GSE55457, and GSE51588 data–sets were merged into an independent data–set after quality control, and then the joint diagnostic efficiency of the obtained diagnostic markers was verified based on this independent data–set. The LASSO algorithm was applied with the “*glmnet*” package [22]. Furthermore, SVM-RFE is a machine learning method based on support vector machine, which is used to find the best variables by deleting SVM-generated eigenvectors. SVM module was established to further identify the diagnostic value of these biomarkers in OA by “*e1071*” package [23]. Ultimately, we combined the genes from either LASSO or SVM-RFE algorithms for further analysis. A two-sided *p* < 0.05 was considered to be statistically significant.

### 2.5. Evaluation of Immune Cell Infiltration

We uploaded the gene expression matrix data to CIBERSORT, filtered out the samples with *p* < 0.05, and obtained the immune cell infiltration matrix. Then, we used “*ggplot2*” package to perform PCA clustering analysis on immune cell infiltration matrix data to draw a two-dimensional PCA clustering map. “*corrplot*” package [24] was used to draw a correlation heatmap to visualize the correlation of 22 types of infiltrating immune cells; “*ggplot2*” package was used to draw violin diagrams to visualize the differences in immune cell infiltration.

### 2.6. Correlation Analysis between Diagnostic Markers and Infiltrating Immune Cells

The “*ggstatsplot*” package (https://github.com/IndrajeetPatil/ggstatsplot) was used to perform Spearman correlation analysis on diagnostic markers and infiltrating immune cells and the “*ggplot2*” package was used to visualize the results.

## 3. Results

### 3.1. Data Preprocessing and DEGs Screening

**Figure 1:**
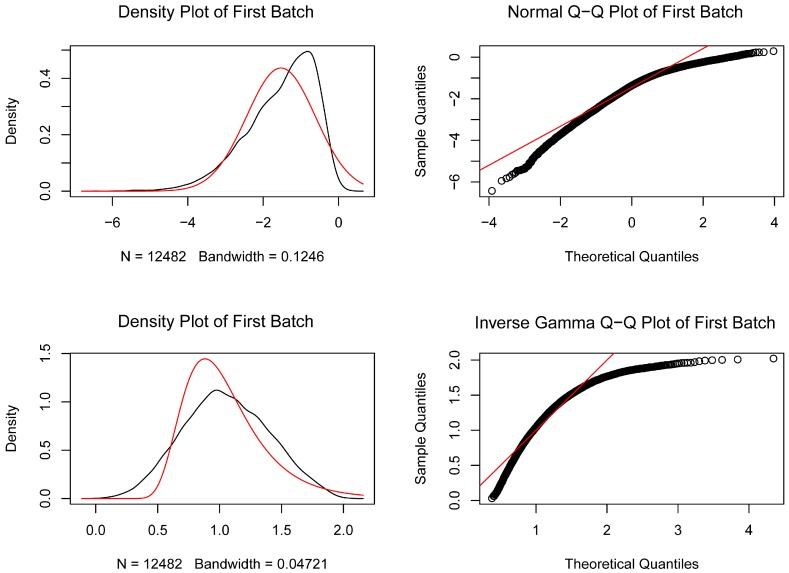
The quantile–quantile (Q-Q) plot of the inter-batch difference of GSE55235 and GSE55457 datasets is removed. The red line represents the density map of the GSE55235 data distribution, the black line represents the density map of the GSE55457 data distribution, and the black circles represent the quantiles corresponding to the same cumulative probability.

**Figure 2:**
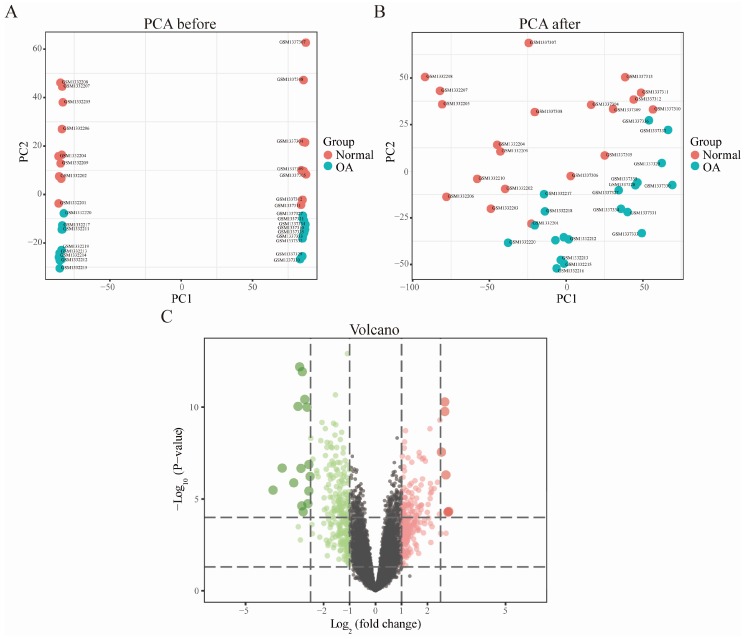
Two-dimensional PCA cluster plot before and after sample correction and volcano map of differentially expressed genes (DEGs). (**A**,**B**) Two-dimensional PCA cluster plot of the GSE55235 and GSE55457 datasets before and after sample correction; blue represents the osteoarthritis (OA) group, and red represents the normal control group. (**C**) Volcano map of DEGs; red represents up-regulated differential genes, grey represents no significant difference genes, and green represents down-regulated differential genes.

First, the inter-batch difference was removed from the gene expression matrix after merging the GSE55235 and GSE55457 datasets, and the Q-Q plot was used to show the effect (Figure 1). The results showed that the inter-batch difference has been removed. The merged gene expression matrix was then normalized and processed, and it is presented in a two-dimensional PCA cluster diagram before and after normalization (Figure 2A,B). The results showed that the clustering of the two groups of samples was more obvious after normalization, indicating that the sample source was reliable. After data preprocessing, we used R software to extract a total of 458 DEGs from the gene expression matrix, as shown in the volcano map (Figure 2C).

### 3.2. Functional Correlation Analysis

**Figure 3:**
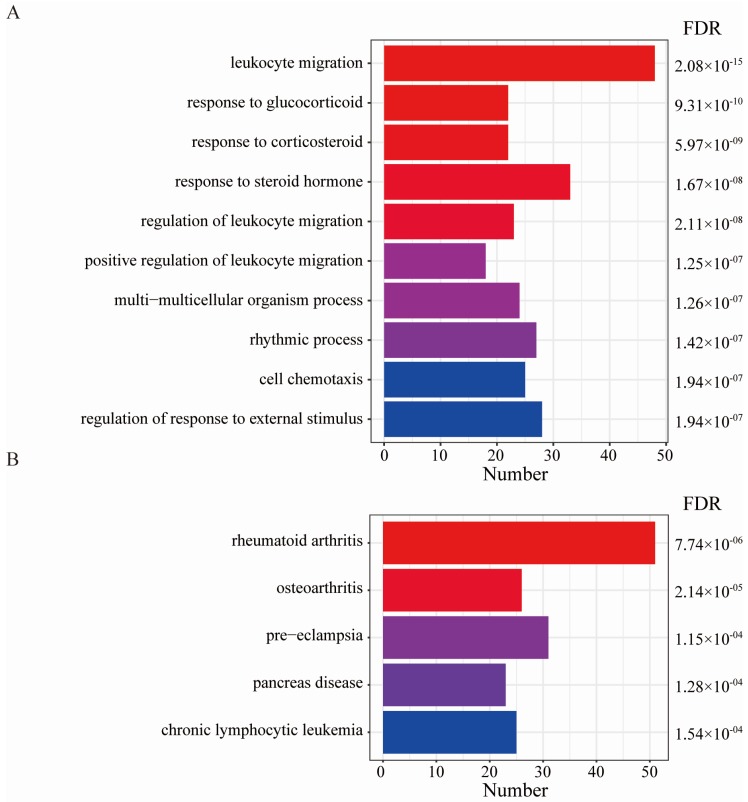
Gene Ontology (GO) and Disease Ontology (DO) enrichment analyses of DEGs. (**A**) GO enrichment analysis, where the horizontal axis represents the number of DEGs under the GO term. (**B**) DO enrichment analysis, where the horizontal axis represents the number of DEGs under the DO term.

**Figure 4:**
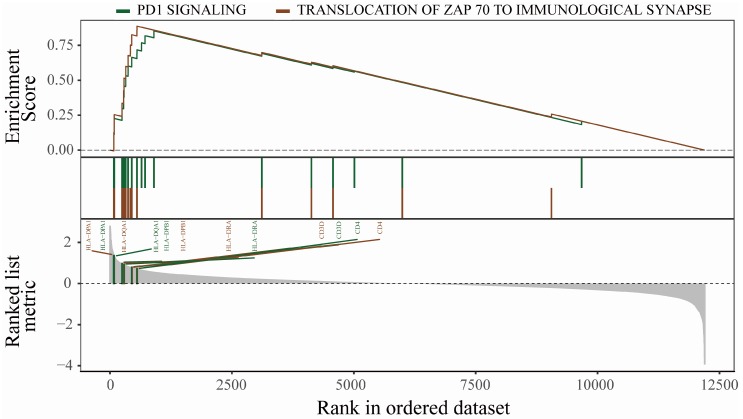
Gene set enrichment analysis. *P*-values were determined using the Kolmogorov–Smirnov test.

GO analysis results showed that DEGs were mainly related to leukocyte migration, positive regulation of response to an external stimulus, cell chemotaxis, and regulation of leukocyte migration (Figure 3A). The DO analysis results are shown in Figure 3B. Diseases enriched by DEGs mainly included chronic lymphocytic leukemia, osteoarthritis, pancreas disease, and rheumatoid arthritis. GSEA results showed that the enriched pathways mainly involved PD1 signaling and the translocation of ZAP-70 to the immunological synapse pathway (Figure 4). HLA−DPA1, HLA−DQA1, HLA−DPB1, HLA−DRA, CD3D, and CD4 played important roles in the signal transduction process of the two pathways. The above results suggest that the immune response plays an important role in OA.

### 3.3. Screening and Verification of Diagnostic Markers

**Figure 5:**
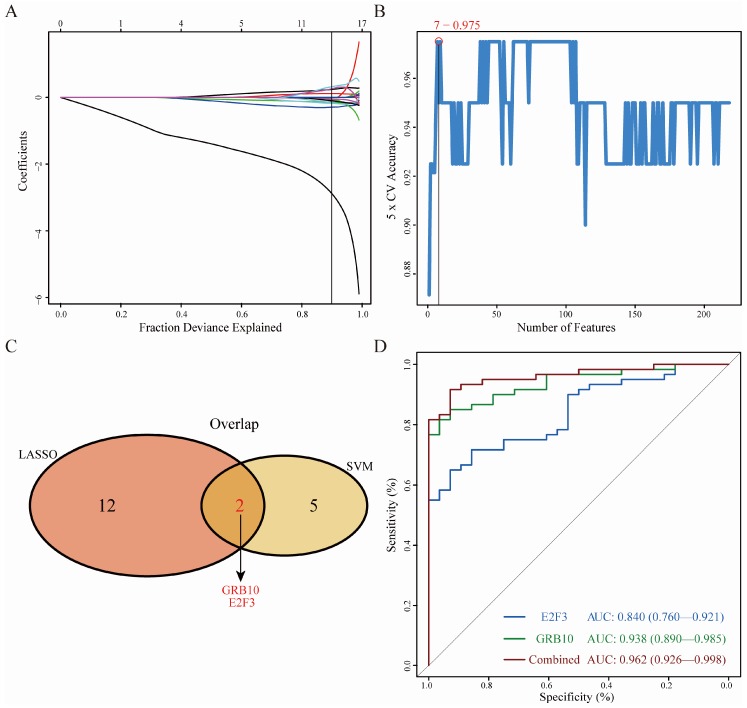
Screening and verification of diagnostic markers. (**A**) Least absolute shrinkage and selection operator (LASSO) logistic regression algorithm to screen diagnostic markers. Different colors represent different genes. (**B**) Support vector machine-recursive feature elimination (SVM-RFE) algorithm to screen diagnostic markers. (**C**) Venn diagram shows the intersection of diagnostic markers obtained by the two algorithms. (**D**) The ROC curve of the diagnostic efficacy verification after fitting two diagnostic markers to one variable.

We used LASSO logistic regression algorithm to identify 14 genes from DEGs as diagnostic markers for OA (Figure 5A); seven genes were identified from DEGs using the SVM-RFE algorithm as diagnostic markers (Figure 5B). The gene markers obtained by the two algorithms were overlapped, and finally two diagnostic related genes were obtained (Figure 5C). In order to further test the diagnostic efficacy of GRB10 and E2F3, we validated it with the GSE51588 dataset as the validation set. When GRB10 and E2F3 were fitted into one variable, the diagnostic efficiency was 1 in the training set and reached a higher level in the validation set (AUC = 0.962) (Figure 5D), indicating that GRB10 and E2F3 had high diagnostic value.

### 3.4. Immune Cell Infiltration Results

**Figure 6:**
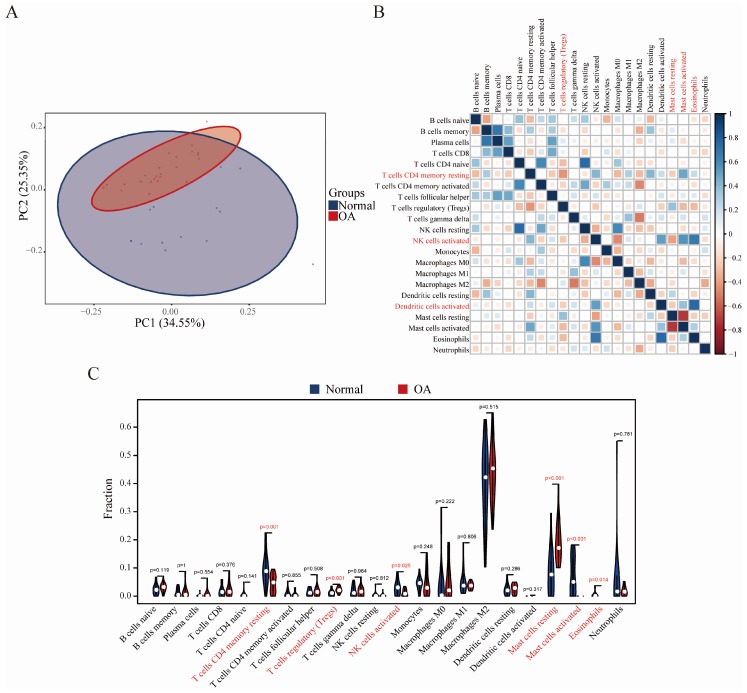
Evaluation and visualization of immune cell infiltration. (**A**) PCA cluster plot of immune cell infiltration between OA samples and control samples. (**B**) Correlation heat map of 22 types of immune cells. The size of the colored squares represents the strength of the correlation; blue represents a positive correlation, red represents a negative correlation. The darker the color, the stronger the correlation. (**C**) Violin diagram of the proportion of 22 types of immune cells. The red marks represent the difference in infiltration between the two groups of samples.

PCA cluster analysis can be used to test the consistency of biological repetition and the difference of different groups. PCA cluster analysis results of immune cell infiltration showed that there was a significant difference in immune cell infiltration between the OA sample and the control sample (Figure 6A). Correlation heatmap of the 22 types of immune cells revealed that activated NK cells, activated dendritic cells, and eosinophils had a significant positive correlation. Resting CD4 memory T cells and activated mast cells also had a positive correlation. Resting CD4 memory T cells had a significant negative correlation with regulatory T cells, and activated NK cells had a negative correlation with resting mast cells (Figure 6B). The violin plot of the immune cell infiltration difference showed that, compared with the normal control sample, regulatory T cells and resting mast cells infiltrated more, while resting CD4 memory T cells, activated NK cells, activated mast cells, and eosinophils infiltrated less (Figure 6C).

### 3.5. Correlation Analysis between GRB10, E2F3, and Infiltrating Immune Cells

**Figure 7:**
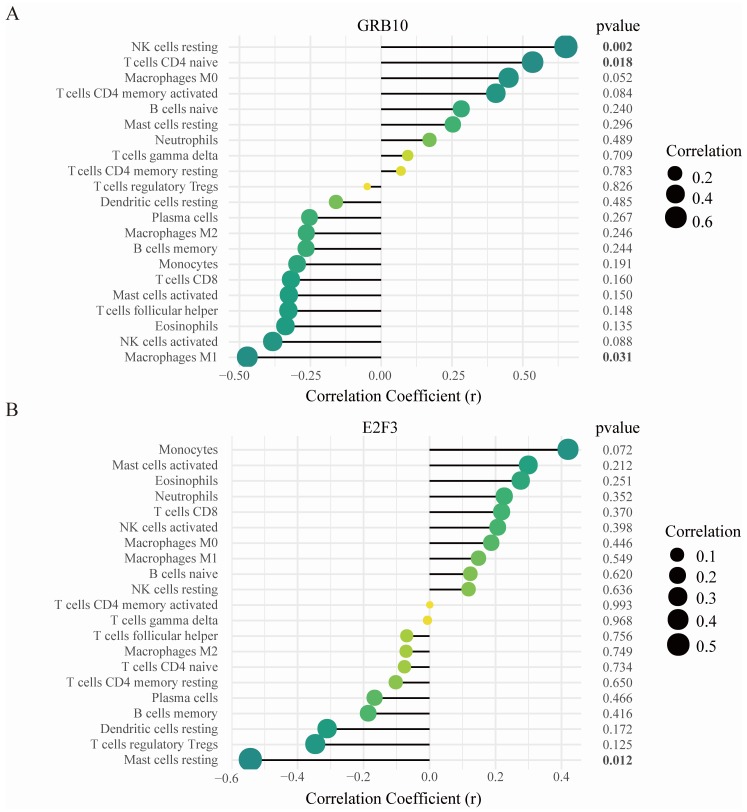
Correlation between GRB10, E2F3, and infiltrating immune cells. (**A**) Correlation between GRB10 and infiltrating immune cells. (**B**) Correlation between E2F3 and infiltrating immune cells. The size of the dots represents the strength of the correlation between genes and immune cells; the larger the dots, the stronger the correlation, and the smaller the dots, the weaker the correlation. The color of the dots represents the p-value, the greener the color, the lower the p-value, and the yellower the color, the larger the p-value. *p* < 0.05 was considered statistically significant.

Correlation analysis showed that GRB10 was positively correlated with resting NK cells (r = 0.642, *p* = 0.002), and naive CD4 + T cells (r = 0.525, *p* = 0.018) and negatively correlated with macrophages M1 (r = −0.482, *p* = 0.031) (Figure 7A); E2F3 was negatively correlated with resting mast cells (r = −0.552, *p* = 0.012) (Figure 7B).

## 4. Discussion

OA is a chronic joint disease that causes cartilage degradation, synovial inflammation, subchondral bone remodeling, and osteophyte formation [25]. Due to the lack of early diagnostic indicators, patients with OA often lose the best opportunity for treatment, resulting in a poor prognosis. In addition, research shows that immune cell infiltration plays an important role in the development of OA [5,6]. Therefore, finding specific diagnostic markers and analyzing the pattern of OA immune cell infiltration have profound significance for improving the prognosis of OA patients. With the rapid development of science and technology, bioinformatics has provided a powerful strategy for screening molecular markers, and CIBERSORT tools have also facilitated the analysis of immune cell infiltration patterns of diseases. In this study, we sought to identify diagnostic markers for OA and further explore the role of immune cell infiltration in OA.

We downloaded the OA expression profile dataset from the GEO database and identified a total of 458 DEGs. GO enrichment analysis showed that DEGs were mainly related to leukocyte migration, positive regulation of response to external stimulus, and cell chemotaxis. The diseases enriched by DO mainly include chronic lymphocytic leukemia, osteoarthritis, and rheumatoid arthritis. The above results suggest that the immune response plays an important role in OA. In addition, the pathway enriched by GSEA mainly involves PD1 signaling and translocation of ZAP-70 to the immunological synapse. Shan et al. [26] showed that the percentage of PD1 in peripheral blood of patients with OA is significantly higher than that of healthy patients, which may play an important role in the progression of OA. The above research results are consistent with our analysis data, suggesting that the analysis results of this study are accurate.

SVM-RFE is a machine learning method based on support vector machines that searches for the best variable by subtracting the feature vectors generated by SVM [20]. LASSO logistic regression is also a machine learning method that determines the variable by finding λ when the classification error is the smallest [21]. The two algorithms are mainly used to screen feature variables and build the best classification model. In this study, GRB10 and E2F3 were identified as diagnostic markers of OA by combining SVM-RFE and LASSO logistic regression methods. Each algorithm has its own inherent characteristics. It should be noted, however, that the GRB10 and E2F3, which were selected by integrating the union of features from LASSO and SVM-RFE, were reliable in further validations in this study, suggesting that the integration strategy was feasible. The encoded product of GRB10 is an adaptor protein that interacts with insulin receptors and insulin-like growth factor-1 receptors participating in processes such as cell proliferation, apoptosis, and migration [27,28]. One study showed that GRB10 is highly expressed in leukemia, and that the level of GRB10 expression is related to the survival of leukemia patients [29]. Grb10 plays a key role in the function of vascular smooth muscle cells by regulating the proliferation, migration, and inflammatory gene expression of these cells [30]. Another study reported that the expression of GRB10 in the hippocampus of diabetic rats is increased, impairing neural function and cognition by negatively regulating the insulin-like growth factor-1 receptor signaling pathway [31]. Moreover, the expression of insulin-like growth factor-1 is related to the proliferation, differentiation, and matrix synthesis of chondrocytes, which are essential in cartilage morphogenesis [32]. Given that GRB10-encoded proteins often interact with insulin receptors and insulin-like growth factor receptors, we believe that GRB10 is likely to be involved in regulating the pathological process of OA. E2F3 is a member of the E2F family and exists in most cell types in the body. It targets retinoblastoma proteins and plays an important role in cell cycle and proliferation [33]. A study has shown that E2F3 plays a key role in muscle and bone development and affects the normal growth of mice [34]. Inhibition of E2F3 expression promotes the development of diabetic nephropathy [35]. Furthermore, E2F3 plays a key role in various biological processes such as lens development, cardiac neovascularization, DNA damage response, and neuronal migration [36,37,38,39]. E2F3 protein is an important participant in the cell cycle process, and an abnormal regulation of the cell cycle is closely related to the development of OA [40]. Therefore, we speculate that E2F3 may play an important role in the disease progression of OA. Evidence from previous studies suggests that GRB10 and E2F3 may be involved in the development of OA and have the potential to be used as diagnostic markers for OA, but numerous clinical studies are still needed to verify the diagnostic value of GRB10 and E2F3.

To further explore the role of immune cell infiltration in OA, we used CIBERSORT to conduct a comprehensive evaluation of OA immune infiltration. We found that an increased infiltration of mast cells, regulatory T cells, and a decreased infiltration of resting CD4 T cells, activated NK cells, and eosinophils may be related to the occurrence and development of OA. Previous studies have shown that the infiltration of mast cells in the OA synovial tissue is relatively high and is related to structural damage in patients with OA [41]. It has also been shown that regulatory T cells are abundant in OA, and their level corelates with inflammatory factor levels [42]. Ezawa et al. [43] found that the accumulation of memory CD4 + T cells is a common phenomenon during the local inflammatory response of joints and may be involved in the formation of chronic OA. Benigni et al. [44] confirmed through in vivo experiments that neutrophils and NK cells play an important role in osteoarthritis, and their interaction is promoted by the CXCL10/CXCR3 axis. The above literature evidence combined with our analysis results have shown that resting mast cells, regulatory T cells, resting CD4 memory T cells, and activated NK cells play important roles in OA and should be the highlight of further studies. However, there is no research conducted on the role of eosinophils in OA, and further experimental data are required. In addition, our results reveal details of infiltration of 22 types of immune cells in OA. Activated mast cells and regulatory T cells infiltration are closely related to resting CD4 memory T cells infiltration; activated NK cells and activated dendritic cells infiltration are closely related to eosinophils infiltration. The specific mechanisms of these correlations require further experimental evidence.

By analyzing the correlation between GRB10, E2F3, and immune cells, it was found that GRB10 was significantly positively correlated with resting NK cells, and naive CD4 + T cells and negatively correlated with M1 macrophages. E2F3 was significantly negatively correlated with resting mast cells. Studies have shown that NK cells and mast cells play important roles in osteoarthritis [41,44]; M1 macrophages polarization in the synovium can aggravate the OA process [45]. Therefore, we speculate that GRB10 raises NK cells and naive CD4 + T cells or reduces M1 macrophages cells and E2F3 reduces mast cells to participate in the occurrence and progress of OA. These assumptions require further research to clarify the complex interactions between genes and immune cells.

We employed novel scientific methods such as SVM-RFE and LASSO logistic regression algorithms to verify OA diagnostic markers and, for the first time, CIBERSORT to analyze immune cell infiltration in OA tissues. However, our research has certain limitations. CIBERSORT analysis is based on limited genetic data that may deviate from heterotypic interactions of cells, disease-induced disorders, or phenotypic plasticity; in addition, our research represents the second mining and analysis of previously published data sets. Although some previous research results are consistent with our analysis results, the reliability of the results of this study needs to be further experimentally validated.

## 5. Conclusions

In conclusion, we found that GRB10 and E2F3 are diagnostic markers of OA. We also found that regulatory T cells, resting mast cells, resting CD4 memory T cells, activated NK cells, and eosinophils may be involved in the occurrence and progress of OA. In addition, GRB10 was significantly associated with resting NK cells, naive CD4 + T cells, and M1 macrophages, and E2F3 was significantly associated with resting mast cells. These immune cells may play a key role in the development of OA, and further exploration of these immune cells may determine the targets of OA immunotherapy and help improve immunomodulatory therapies for OA patients.
